# Improved GOA-based fuzzy PI speed control of PMSM with predictive current regulation

**DOI:** 10.1371/journal.pone.0318094

**Published:** 2025-01-30

**Authors:** Taochang Li, Ang Li, Limin Hou

**Affiliations:** Faculty of Electrical and Control Engineering, Liaoning Technical University, Huludao, Liaoning, China; Ghani Khan Choudhury Institute of Engineering and Technology, INDIA

## Abstract

To address the susceptibility of conventional vector control systems for permanent magnet synchronous motors (PMSMs) to motor parameter variations and load disturbances, a novel control method combining an improved Grasshopper Optimization Algorithm (GOA) with a variable universe fuzzy Proportional-Integral (PI) controller is proposed, building upon standard fuzzy PI control. First, the diversity of the population and the global exploration capability of the algorithm are enhanced through the integration of the Cauchy mutation strategy and uniform distribution strategy. Subsequently, the fusion of Cauchy mutation and opposition-based learning, along with modifications to the optimal position, further improves the algorithm’s ability to escape local optima. The improved GOA is then employed to optimize the contraction-expansion factor of the variable universe fuzzy PI controller, achieving enhanced control performance for PMSMs. Additionally, to address the high torque and current ripple issues commonly associated with traditional PI controllers in the current loop, Model Predictive Control (MPC) is adopted to further improve control performance. Finally, experimental results validate the effectiveness of the proposed control scheme, demonstrating precise motor speed control, rapid and stable current tracking, as well as improved system robustness.

## 1. Introduction

The Permanent Magnet Synchronous Motor (PMSM) has attracted significant attention due to its compact structure, high efficiency, rapid dynamic response, and exceptional speed control performance [1]. In recent years, the application of PMSMs has expanded rapidly across various fields, including industrial automation, electric transportation, renewable energy systems, and other sectors, largely driven by advancements in permanent magnet material technologies. However, like other electric motors, PMSMs face challenges related to parameter uncertainties and load disturbances during practical operation. Conventional fixed-parameter Proportional-Integral-Derivative (PID) control strategies often fall short of meeting the stringent requirements of modern industrial control systems in terms of performance and robustness [2,3].

Consequently, numerous innovative techniques have been proposed by researchers to improve the control performance of PMSMs. These methods include fuzzy control [4,5], model predictive control [6,7], sliding mode control [8,9], among others. Additionally, there has been increasing interest in leveraging intelligent algorithms and neural networks in the control of PMSM systems, as evidenced by recent studies [10–17]. For instance, in reference [18], an adaptive fuzzy PI controller is introduced for the vector control system of PMSMs. This approach dynamically adjusts the proportional and integral parameters based on the system error and error rate, thereby effectively enhancing the control performance of PMSMs. However, conventional fuzzy PI control methods often operate within fixed domains, which limits their ability to accommodate complex and dynamic system behaviors. To address this limitation, reference [19] proposes a particle swarm optimization (PSO)-based fuzzy PI control method as a parameter optimization strategy. In this method, the proportional factor and quantization factor of the fuzzy PI controller are optimized through iterative updates of the PSO algorithm, resulting in improved convergence accuracy. This approach enhances the response speed and robustness of PMSMs under varying operational conditions.

In a rigorous examination of motor control methodologies, reference [20] investigates the application of the Extreme Learning Machine (ELM) technique to enhance the dynamic performance and robustness of the system by accurately estimating system disturbances. Building upon this, reference [21] introduces a novel approach that integrates the ELM with the Artificial Bee Colony (ABC) algorithm. This hybrid technique is applied within the PMSM fuzzy PI speed control system, yielding significant reductions in settling time and marked improvements in steady-state accuracy. Nonetheless, while intelligent algorithms and neural networks present promising avenues for advancing motor control, they often encounter challenges such as parameter tuning and algorithm convergence. These issues increase the complexity of implementing such methodologies in practical applications [22].

In summary, this manuscript proposes an enhanced GOA-based fuzzy PI speed control strategy for PMSMs, incorporating predictive current regulation to optimize the performance of high-efficiency PMSM speed control systems. The key contributions of this study are summarized as follows:

(1) This study introduces an improved control framework that integrates an enhanced variable domain fuzzy PI controller with a model predictive current controller (MPCC) in a cascaded structure to achieve high-performance speed regulation for PMSMs.
(2) An enhanced GOA is developed by incorporating mixed Cauchy variation and uniform distribution to address the issues of premature convergence and local optima. Furthermore, a variable domain fuzzy PI speed controller is designed for PMSMs, enabling dynamic adjustments of the fuzzy control domain through the improved GOA, thereby enhancing control flexibility and robustness.

The structure of this manuscript is organized as follows: Section 2 presents the design of the improved GOA-based fuzzy PI speed controller for PMSMs. Section 3 introduces the model predictive current control methodology. Simulation and experimental results are discussed in Section 4. Finally, the conclusions of the study are summarized in Section 5.

## 2. Improved GOA-based fuzzy PI speed controller design for PMSM

### 2.1. Mathematical model of PMSM

The primary objective of this study revolves around a surface-mounted PMSM. Specifically, the mathematical representation of the motor within the d-q coordinate system is presented as (1).


$$\{\begin{array}{l} u_{d}=R_{s}i_{d}+L\frac{di_{d}}{dt}-\omega_{e}Li_{q}\\ u_{q}=R_{s}i_{q}+L\frac{di_{q}}{dt}+\omega_{e}(Li_{d}+\psi_{f}) \end{array}$$ (1)

where *u*_*d*_ and *u*_*q*_ are the stator voltage, while *i*_*d*_ and *i*_*q*_ are stator current in the d-q frame, *R*_*s*_ is stator resistance, *L* is the stator inductance, *ω*_*e*_ is the electric angular velocity, *ψ*_*f*_ is permanent magnet flux linkage.

### 2.2. Variable domain fuzzy PI control

**Fig 1:**
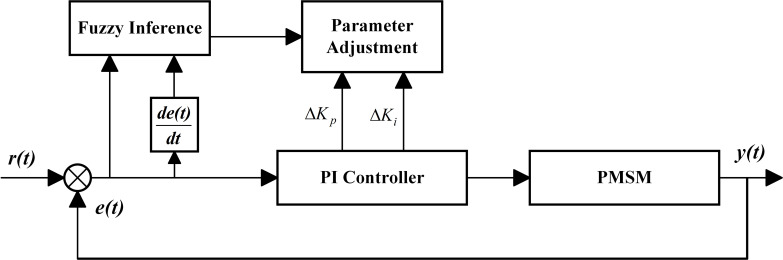
Schematic diagram of Fuzzy PI controller.

The conventional PI controllers have a limitation in achieving online parameter tuning, resulting in the inability to adapt to different operating conditions and hindering the improvement of their dynamic performance. To overcome this limitation and enhance the system’s dynamic performance and disturbance rejection ability, a fuzzy PI self-tuning controller is developed by integrating the principles of fuzzy control theory with the conventional PI strategy, as illustrated in Fig 1.

The conventional fuzzy PI control method involves the continuous monitoring of the system’s states, and then computes the velocity error *e* and its rate of change *e*_c_ within the motor system, which are then subjected to fuzzification. utilizing the pre-designed fuzzy rules, a fuzzy inference is carried out. The next step is defuzzification, which converts the fuzzy quantities into precise values. Subsequently, this process effectively enables real-time adjustment of the PI controller’s parameters. The variables *e*, *e*_c_, *ΔK*_*p*_ and *ΔK*_*i*_ make use of the identical fuzzy subsets {NB, NM, NS, ZO, PS, PM, PB}.

However, the arrangement of fuzzy rules heavily depends on user expertise. While a large number of rules can accurately depict system outputs, it also introduces complexity into the system. On the other hand, a limited number of rules may simplify the system, but it may compromise the effectiveness of control.

**Fig 2:**
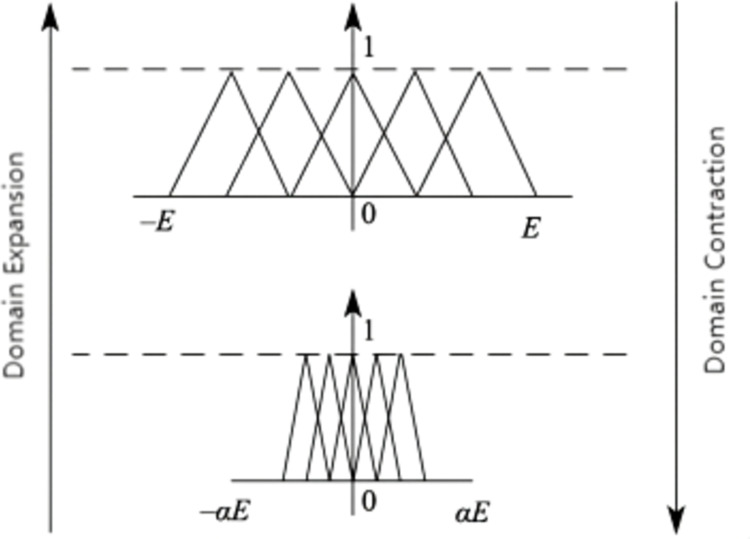
Schematic diagram of variable domain principle.


The variable domain fuzzy PI does not necessitate extensive domain-specific expert knowledge. Instead, it only requires a general understanding of the rule trends. The main concept behind this approach is to dynamically adjust the domain of the fuzzy controller by utilizing a scaling factor. The scaling factor’s variation is directly proportional to the variable deviation. Fig 2 presents a schematic diagram illustrating this concept, where *α* represents the scaling factor, and [*E*, - *E*] represents the domain of the fuzzy controller. As the input to the controller increases or decreases, the scaling factor adjusts accordingly. Currently, there is no standardized form for the scaling factor in variable domain fuzzy control algorithms. Typically, the scaling factor of the domain is designed based on a function as shown:

$$\alpha (x)=1-\lambda e^{-kx^{2}},\lambda \in (0,1)$$ (2)

where *k* > 0 is the adjustment coefficient of the expansion factor.

In light of the challenges associated with determining the optimal values for parameter, the paper employs an improved GOA to optimize the scaling factor. Consequently, a variable domain fuzzy PI controller is developed based on the improved GOA algorithm. The domains of the input and output variables are defined as shown:


$$\{\begin{array}{c} X_{E}\\ X_{E_{c}} \end{array}\begin{array}{c} =\\ = \end{array}\begin{array}{c} \left[-\alpha (e)E,\alpha (e)E\right]\\ \left[-\beta (e_{c})E_{c},\beta (e_{c})E_{c}\right] \end{array}$$ (3)

### 2.3. Improved GOA-based variable domain fuzzy PI control

The GOA is an innovative heuristic algorithm that draws inspiration from nature. It mimics the behavior of grasshoppers, where the adult grasshoppers conduct a wide-scale search while the nymphs engage in small-scale movements, both ultimately converging towards a food source. This corresponds precisely to the global exploration and local exploitation aspects of heuristic algorithms. The process of the grasshopper swarm searching for resources is viewed as the process of seeking the optimal solution [23].

During the exploration phase, it is essential for the algorithm to exhibit strong random behavior in order to thoroughly explore the entire search space. Moreover, the initial solutions should demonstrate a wide range of variations, thereby increasing the likelihood of uncovering the region where the optimal solution is expected to reside. Subsequently, in the exploitation phase, the algorithm narrows down the search space, leading to smaller variations in the search process, thus facilitating a more refined exploration. Furthermore, achieving an appropriate balance between the exploration and exploitation phases through the utilization of an improved GOA algorithm is crucial for effectively identifying the optimal solution to a given optimization problem. This approach helps to address the limitations of the original GOA, including its vulnerability to local optima and premature convergence. The specific implementation process involves the following steps.

1) Initialize the population space size N, space dimension, maximum iteration count *T*_*max*_, as well as parameters *c*_*max*_ and *c*_*min*_. Additionally initialize the population positions *X*_*i*_*i* = (1, 2 … *N* ).
2) Designing fitness functions. In the context of the PMSM speed control system, the objective is to develop a fitness function that effectively reduces both overshoot and rise time. To achieve this simultaneous optimization, the fitness function can encompass the integration of the absolute value of the error as the primary performance indicator. Moreover, to ensure that the control input remains within acceptable limits, a squared term of the control input is incorporated into the fitness function. This results in the following fitness function Eq (4).

$$F=\omega_{1}\int_{0}^{\infty }\tau \left|e(\tau )\right|d\tau +\omega_{2}\int_{0}^{\infty }\tau u^{2}(\tau )d\tau$$ (4)

where *ω*_1_ and *ω*_2_ are the weight coefficients for the cumulative speed deviation and the cumulative input square, and *ω*_1_ + *ω*_2_ = 1_._

The setting of these weights is grounded in empirical considerations, and the ultimate determination of weights for the objective fitness function is achieved through experimentation involving various combinations of weights.

3) Calculate individual fitness. The optimal value is stored in the target value *T*_*d*_. Furthermore, the parameters *c* and positions of the grasshoppers are to be updated. The calculation formulas for the conventional GOA are denoted by Eq (5) and (6).

$$c=c_{\max}-t\frac{c_{\max}-c_{\min}}{T_{\max}}$$ (5)

$$X_{i}^{d}=c(\sum_{j=1,j\neq i}^{N}c\frac{ub_{d}-lb_{d}}{2}s(\left|x_{j}^{d}-x_{i}^{d}\right|)\frac{x_{j}-x_{i}}{d_{ij}})+T_{d}$$ (6)

where and are the maximum and minimum values of the adaptive parameter. is the current iteration count. is the position of the *i* − *th* grasshopper in the *d* − *th* dimension. and are the upper and lower bounds of the *d*-dimensional variable. *T*_*d*_ is the target position. *s* is the inter-individual impact function. *d*_*ij*_ is the distance between the *i* − *th* grasshopper and the *j* − *th* grasshopper in the *d* − *th* dimension. and *c* is the key to coordinating exploration.

According (5), it can be seen that the function value decreases with the iteration count, which does not meet the practical exploration and adaptation optimization process. Therefore, this paper introduces a random number γ following a uniform distribution to dynamically improve the variation of *c*. By introducing the uniform distribution number γ through the improved function, early-stage exploration can be more comprehensive, and at the same time, it accelerates the convergence speed of the algorithm in the later stages. The ability to modify the parameters dynamically facilitates the acceleration of algorithm development. The Eq (5) is rewritten as (7).


$$c=\gamma {\left(\mathrm{lg}\frac{c_{\max}}{T_{\max}}\right)}^{-20t}+\exp{\left(\frac{t}{T_{\max}}\right)}^{-20(c_{\max}-c_{\min})}$$ (7)

4) Using the Cauchy operator and a segmented approach. The process of updating individual positions is accomplished using the Cauchy operator and a segmentation approach. During the initial stage of the algorithm, the optimal solution of each generation is stored in memory, and the Eq (6) is enhanced as (8) for better performance.

$$X_{\text{new}}(t+1)=a_{1}\times W+a_{2}\times r_{1}(X_{best}(t)-X_{i}(t))+a_{2}\times r_{2}(X_{j}(t)-X_{k}(t))$$ (8)

where *X*_*new*_ (*t* + 1) is the position of the *i* − *th* grasshopper in the (*t* + 1) − *th* generation. *X*_*best*_ (*t*) is the optimal position in the *t* − *th* generation. *X*_*j*_ (*t*)  and *X*_*k*_ (*t*)  are two random positions in the *t* − *th* generation. *r*_1_ and *r*_2_ are two random numbers generated in the range [0,1]. *a*_1_ is the memory coefficient. *a*_2_ is the information exchange coefficient.


$$a_{1}=\exp{\left(c(t)-30\frac{t}{T_{\max}}\right)}^{-t}$$ (9)

$$a_{2}={\left(\frac{T_{\max}-t}{T_{\max}}\right)}^{N}$$ (10)

$$W=\sum_{j=1,j\neq i}^{N}c\frac{ub_{d}-lb_{d}}{2}s(\left|x_{j}^{d}-x_{i}^{d}\right|)\frac{x_{j}-x_{i}}{d_{ij}})$$ (11)

The enhanced algorithm incorporates the entirety of position information, thereby facilitating the exchange of information among individuals. Furthermore, the exploration aspect is expanded through the random selection of two individuals to spearhead the position update. The latter half of the algorithm introduces the Cauchy operator for the mutation operation, enabling the algorithm to break free from local optima, preventing premature convergence and accelerating the convergence rate. The formula is as follows.


$$X_{i}^{d}(t+1)=X_{i}^{d}(t)+Cauchy⊕(X_{best}^{d}(t)-X_{k}^{d}(t))$$ (12)

where Cauchy is the Cauchy operator. $X_{k}^{d}(t)$ is the random position of the *k* − *th* grasshopper in the *d* − *th* dimension. The algorithm update is determined by a random probability *P*_1_. When *P*_1_ is less than *T*_max_ / 2 individual position updates are performed by using Eq (8), (9), (10), and (11). Otherwise, updates are done by using Eq (12).

5) Incorporating Cauchy mutation. The introduction of Cauchy mutation serves as an active mechanism to perturb and update the target position in order to prevent the algorithm from being confined to local optima.

$$X_{\text{new}}(t+1)=C\text{auchy}⊕X_{\text{best}}(t)$$ (13)

where *X*_*new*_ (*t* + 1) is the target solution for the *t*  + 1 generation.

In order to guarantee the superiority of the new position in comparison to the target position, a greedy mechanism is implemented subsequent to the position update operation. This mechanism involves the evaluation of the fitness of both the new and old target positions, enabling the determination of whether the position should be updated. This approach ensures that the algorithm progresses towards the desired position.

6) Selecting operation. In order to ascertain if the individual fulfills the termination condition, it is necessary to determine whether this condition is met. If indeed it is, the global optimal solution should be outputted. On the other hand, if the condition is not met, it is necessary to repeat steps 3) to 5) until the algorithm reaches the maximum number of iterations.

**Fig 3:**
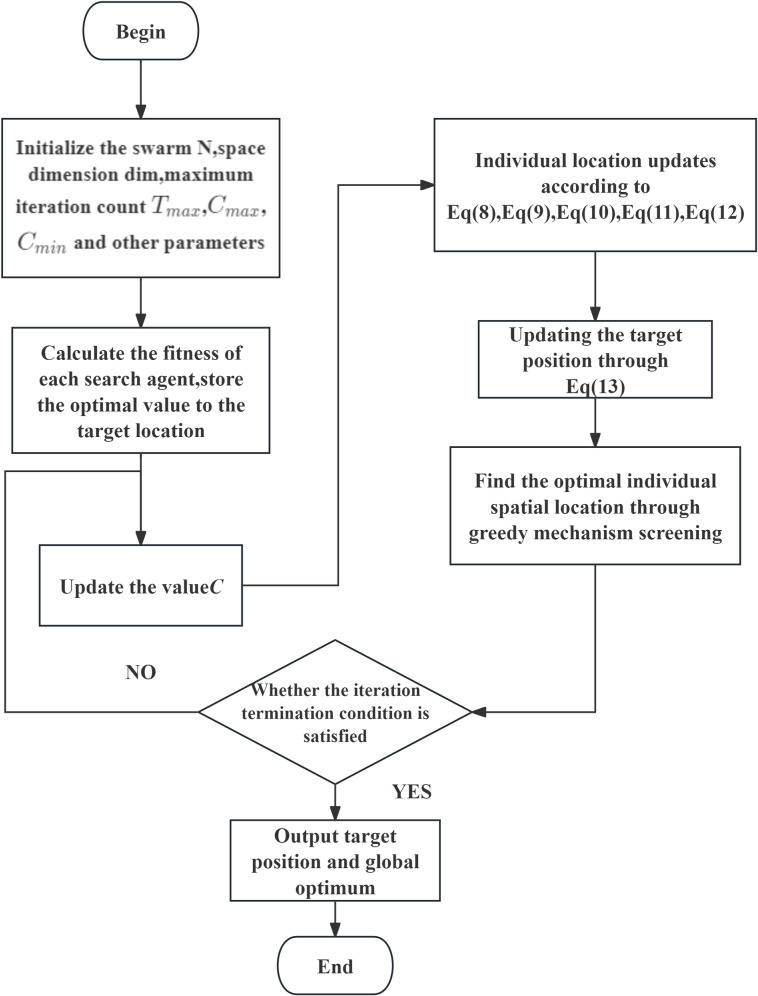
Flowchart of the proposed improved GOA.

The flowchart of the proposed improved GOA is shown in Fig 3.

**Fig 4:**
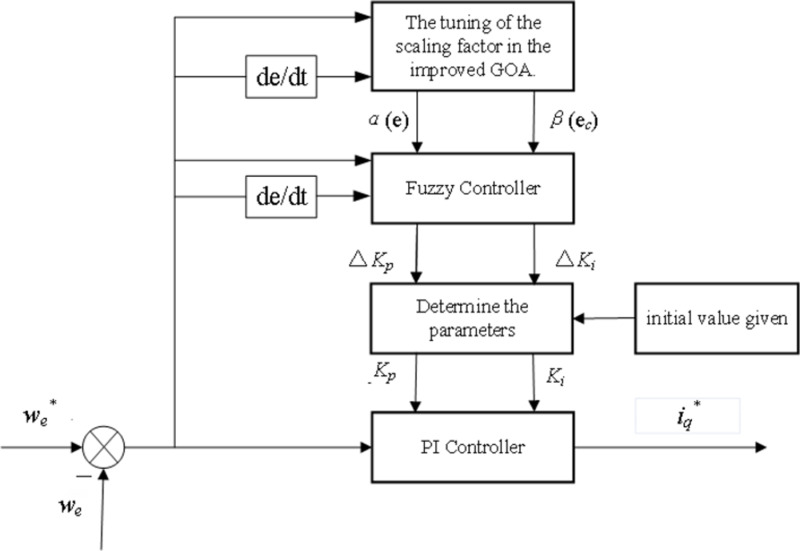
Schematic diagram of improved GOA-based Fuzzy PI controller.

By incorporating the improved GOA with the variable-domain fuzzy PI controller, the scaling factor is dynamically modified according to the input variables and their rates of change. This leads to the generation of more appropriate PI parameter adjustments, which are then combined with the predefined PI parameters. Finally, these optimized parameters are fed into the controller to complete the process of parameter optimization. In the speed control loop of PMSM, the schematic representation of the variable-domain fuzzy PI controller model, which is based on the improved GOA, is depicted in Fig 4.

## 3. Model predictive current control

In conventional vector control systems, the utilization of a PI controller in the current loop gives rise to certain challenges, including sluggish dynamic response and elevated current harmonics. To overcome these limitations, this scholarly paper adopts the MPC approach in the current loop instead of the conventional PI controller, aiming to attain enhanced control performance. By regarding the motor current as state variables, Eq (1) can be altered as (14).


$$\{\begin{array}{l} \frac{di_{d}}{dt}=u_{d}/L-R_{s}{\text{i}}_{\text{d}}/L+\omega_{e}i_{q}\\ \frac{di_{q}}{dt}=u_{q}/L-R_{s}{\text{i}}_{\text{q}}/L-\omega_{e}\psi_{f}/L-\omega_{e}i_{d} \end{array}$$ (14)

In practical systems, it becomes challenging to avoid calculation delays due to the impact of hardware and numerical control. To ensure that the system control performance remains unaffected, it becomes imperative to incorporate a compensation mechanism with a delay of one sample. By discretizing Eq (14) with a sufficiently short sampling time, we can derive the equation for compensating the current with a one-sample delay as presented in (15).


idp(k+1)=(1−TsRsL)id(k)+Tsωeiq(k)+TsLud(k)iqp(k+1)=(1−TsRsL)iq(k)−Tsωeid(k)+TsLuq(k)−TsωeψfL (15)

The compensating current acquired is utilized to substitute the current within the existing prediction model. Consequently, with the integration of one-sample delay compensation, the current prediction model is subsequently revised as (16).


idp(k+2)=(1−TsRsL)id(k+1)+Tsωeiq(k+1)+TsLud(k+1)iqp(k+2)=(1−TsRsL)iq(k+1)−Tsωeid(k+1)+TsLuq(k+1)−TsωeψfL (16)

The objective of the one-step ahead model predictive current control (MPCC) is to minimize the tracking error at the subsequent sampling point. Consequently, the cost function for the conventional MPCC is expressed as:


$$J={\left(i_{d}^{*}-i_{d}^{p}(k+2)\right)}^{2}+{\left(i_{q}^{*}-i_{q}^{p}(k+2)\right)}^{2}$$ (17)

The cost function is minimized by the prediction current, which in turn corresponds to the optimal voltage vector. This optimal voltage vector represents the most desirable output at the current time.

## 4. Simulation and Experiment Results

### 4.1. Simulation Verification

**Fig 5:**
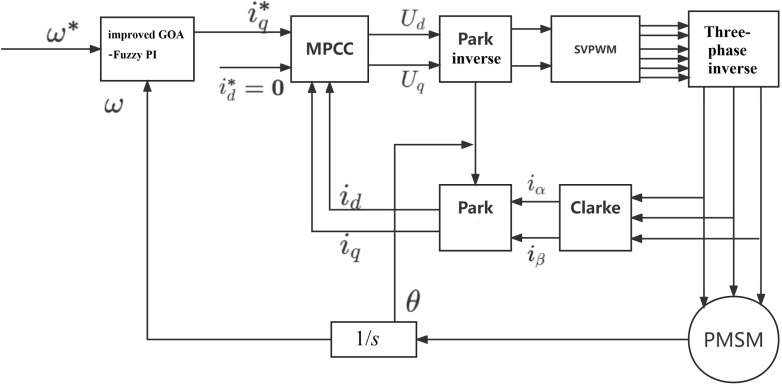
Schematic diagram of PMSM control system.

The framework for the control strategy of the PMSM speed control system, as presented in this research, is depicted in Fig 5. In order to validate the effectiveness of the proposed strategy, simulations were conducted within the Simulink environment. The study involved a comparative analysis of three control strategies in regards to load variations, speed adjustments, and rotation direction changes. Scheme 1, referred to as Fuzzy PI, employs the conventional fuzzy PI for speed control and traditional PI control for current control. The parameters of PI controller are *K*_*P*_ = 30, *K*_*i*_ = 500. Scheme 2 is the model predictive current control strategy of PMSM with GOA variable universe fuzzy PI speed controller. Scheme 3, known as improved GOA-Fuzzy PI, utilizes the control method proposed in this paper.

The simulation experiment employs the GOA algorithm with predetermined parameters. These parameters include a population size of *N* = 30, a maximum iteration count of *T*_max_ = 100, and the variables to be optimized is *d* = 4, *c*_*max*_ = 1 and *c*_*min*_ = 2 × 10^−5^. Moreover, the weights in the fitness function are *ω*_1_ = 0.9 and *ω*_2_ = 0.1.

**Fig 6:**
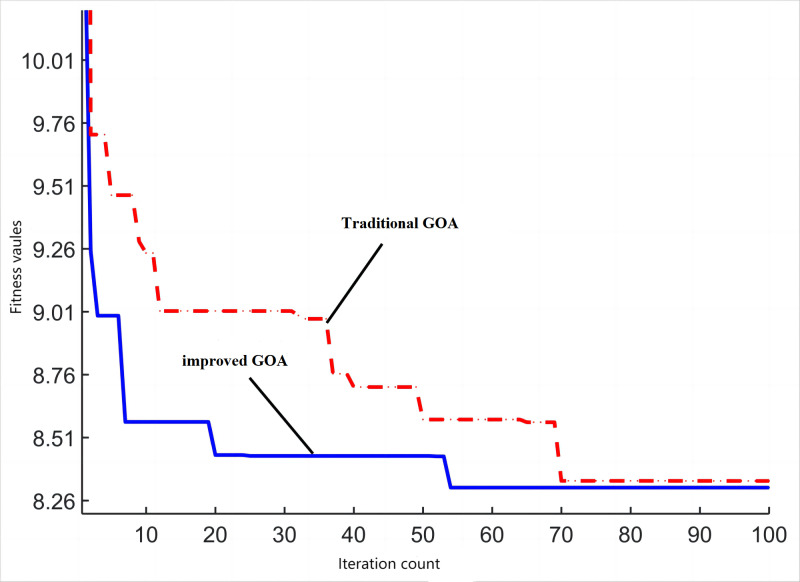
The comparison chart of algorithm fitness values.

The comparison chart depicted in Fig 6 presents the algorithm fitness values at 800 r/min under load. It becomes evident that the improved version of the GOA displays notable improvements in terms of both convergence speed and optimization capability when compared to the conventional GOA.

The parameters of PMSM, which are used in Simulation, are listed in Table 1.

**Table 1:** Operation parameters of PMSM.

Parameters	Value
Stator resistance	0.346*Ω*
Stator inductance	7.8mH
Viscous coefficient	0.005N·s/m
Moment of inertia	0.089*kg* ⋅ *m*^2^
Parameters magnet flus linkage	0.51825Wb
Number of pole pairs	2
Rated power	10kw
Rated voltage	260V
Rated torque	63N·m
Rated speed	1500r/min

**Fig 7:**
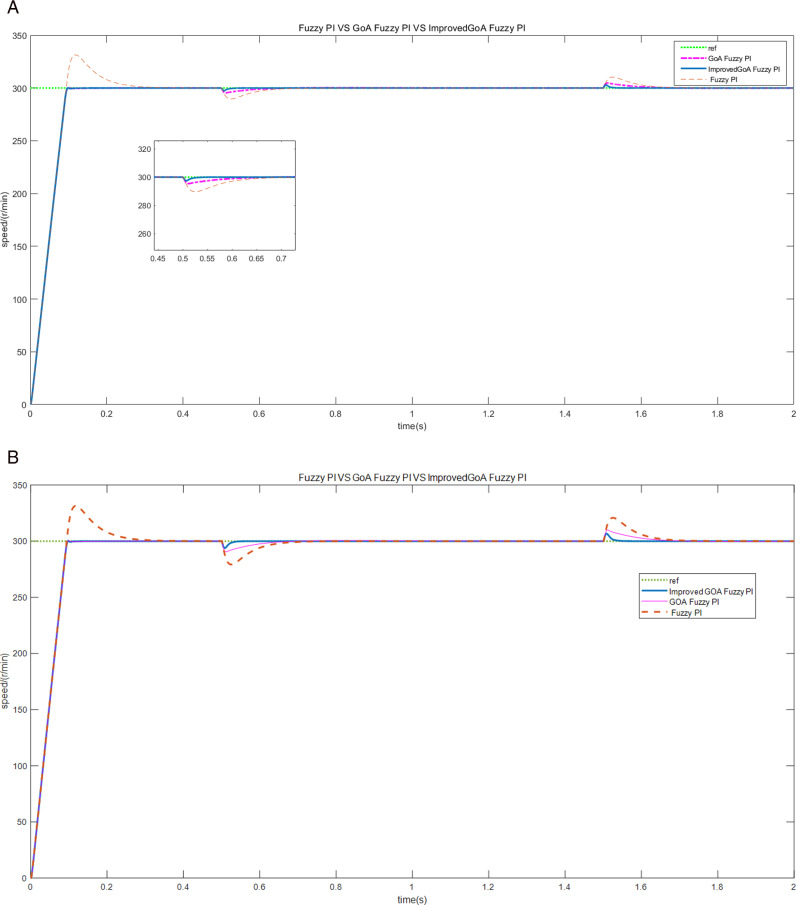
The motor speed curves of load increase/decrease (A) the load of 10 N • m (B) the load of 20 N • m.

**Fig 8:**
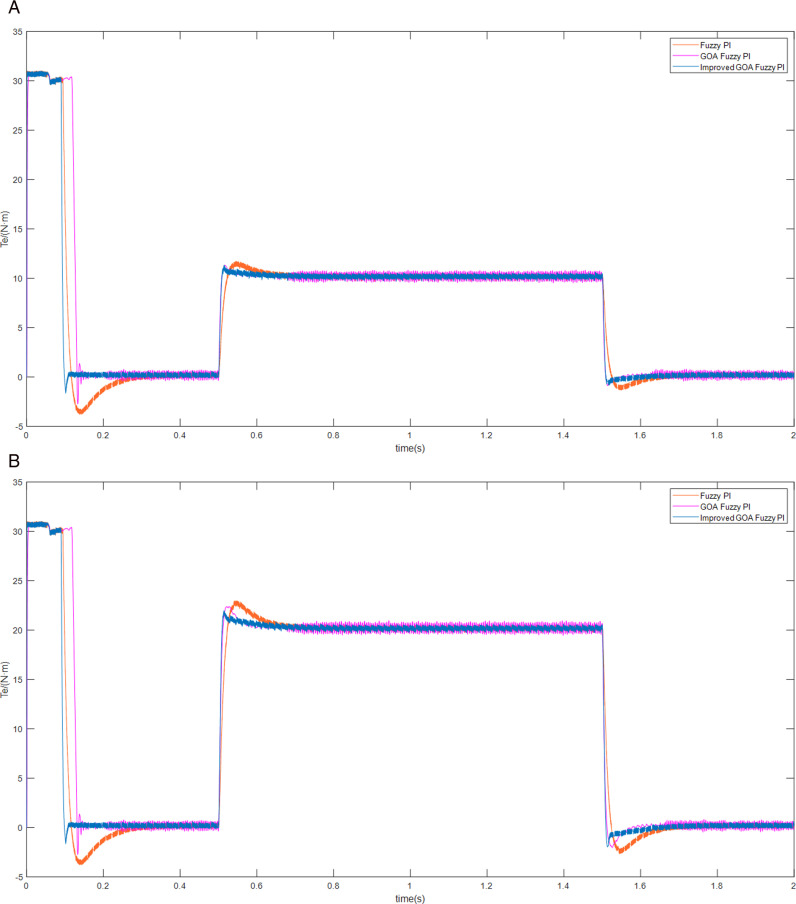
The motor torque curves of load increase/decrease (A) the load of 10 N • m (B) the load of 20 N • m.

**Fig 9:**
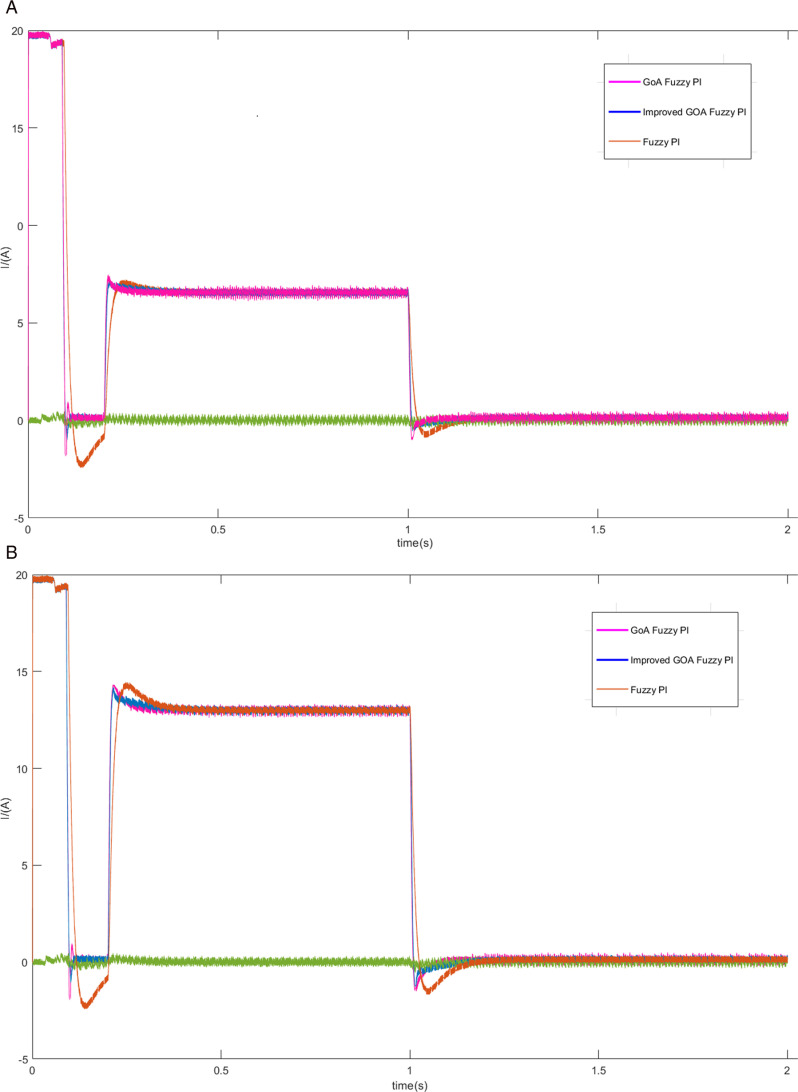
The d-q axis current curves (A) the load of 10 N • m (B) the load of 20 N • m.

Firstly, the motor is started without any external load, operating at a prescribed speed of 300 r/min. Subsequently, the load of 10 N • m is imposed upon the motor at 0.5 seconds. And then the load is suddenly reduced at 1.5 seconds. The experiment was then repeated by changing the load to 20 N • m. The speed, torque and current curves for three schemes are shown in Figs 7–9.

Based on the findings depicted in Fig 7A and 7B, when the given speed is 300 r/min, scheme 1 produces about 10.3% overshoot in the start-up stage, scheme 2 and scheme 3 start smoothly without obvious overshoot. However, the rise time of scheme 2 is about 0.1s, while scheme 3 is only 0.08s, which is better in terms of rapidity. Moreover, scheme 3 has less speed overshoot and quickly returns to a steady state when dealing with different loads.

As can be seen from Fig 8, the motor torque is stabilized at 0 N • m faster because of scheme 3, and the time used is approximately 0.1 s, whereas the torque achieved using the traditional Fuzzy PI strategy requires 0.2 s to reach a stable state. In addition to this, scheme 3 enables the motor to achieve faster and consistent torque. Combined with Fig 9, it can be seen more intuitively that scheme 3 can track the change of current faster at the moment of startup and load change, and the current fluctuation is relatively small.

**Fig 10:**
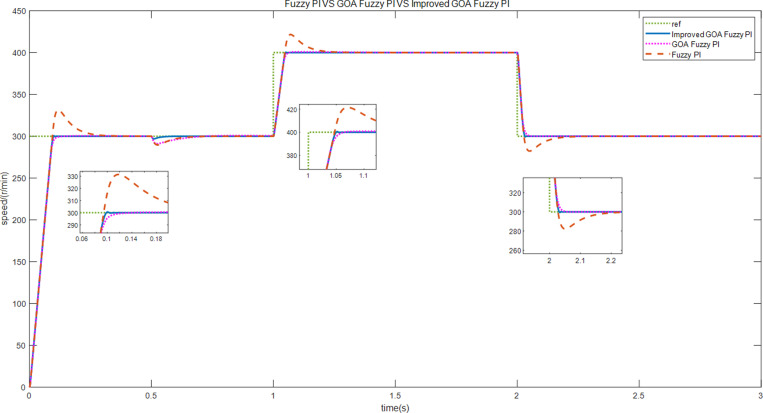
The speed curves of motor speed adjustments.

**Fig 11:**
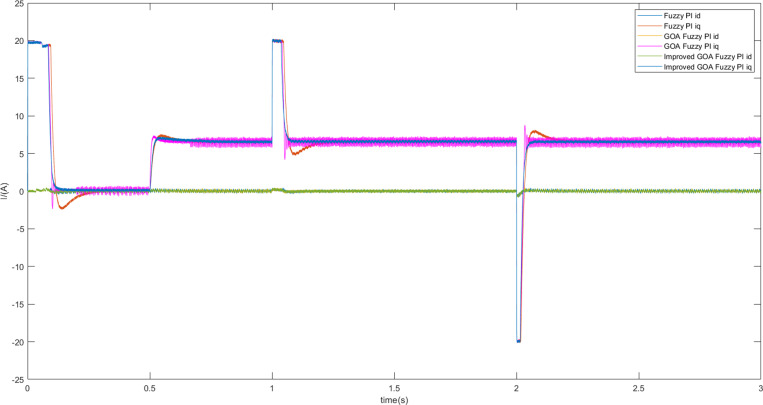
The d-q axis current curves of motor speed adjustments when the motor is loaded with torque.

Three control strategies were simulated under different working conditions. First, the motor starts running at a constant speed of 300 r/min and no-load torque. Then, the motor is loaded with a constant load torque of 10 N • m at 0.5 s. Finally, at 1 s, the given speed changes suddenly, and the speed is changed from 300 r/min to 400 r/min, but is then readjusted to 300 r/min at 2s.The steady-state simulation experiment was carried out under the above conditions. Figs 10 and 11 show the speed and current curves for the three control schemes. Upon comparing the Fig 10, it is evident that scheme 1 displays an overshoot of around 6.8% during the acceleration and deceleration stages, whereas scheme 3 smoothly and rapidly adjusts the speed. From Fig 11, it can be seen the current fluctuation of the q-axis of scheme3 is small, and its operation is stable.

**Fig 12:**
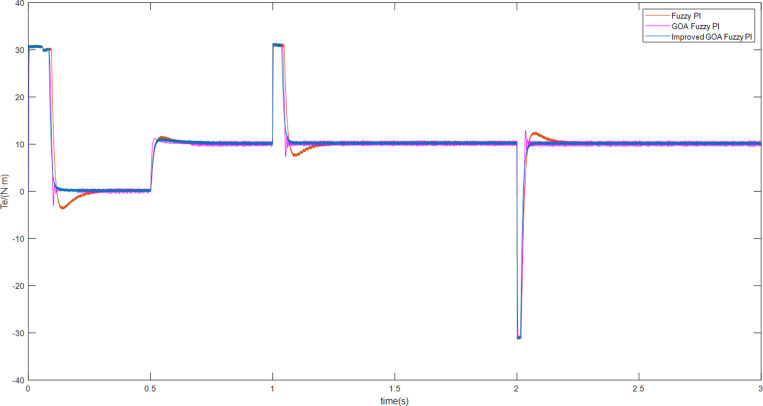
The torque curves of motor speed adjustments.

As can be seen from Fig 12, the torque in the scheme3 system becomes 31 N • m and then returns to the load torque value of 10 N • m after about 0.05 s. On the other hand, in the Fuzzy PI system, the torque returns to the load torque value of 10 N • m, the value of the torque increases to 31 N • m and remains there for 0.1 s. In contrast, the current control system predicted by the model has a better immunity to interference.

**Fig 13:**
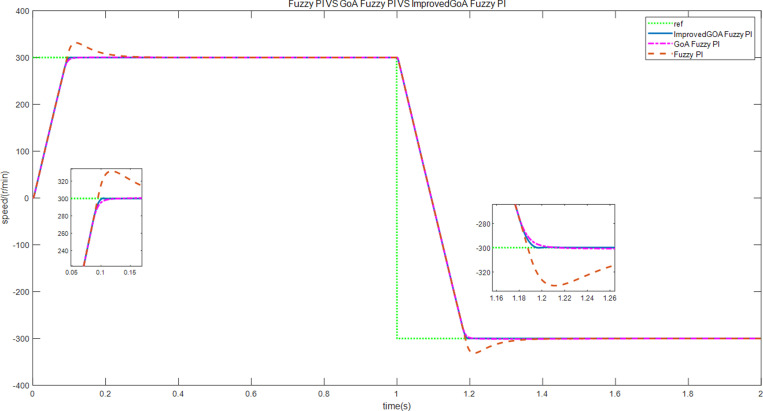
The speed curves of forward and reverse rotation.

At the initial stage, the motor operates at a prescribed speed of 300 r/min. However, after 1 second, the motor speed is altered to -300 r/min. The graphical representation in Fig 13 depicts the speed curves for both forward and reverse rotation under the three schemes. The graph clearly illustrates that scheme 3 rapidly and precisely adapts to changes in speed, thus substantiating the effectiveness of the proposed approach.

### 4.2. Experiment Verification

**Fig 14:**
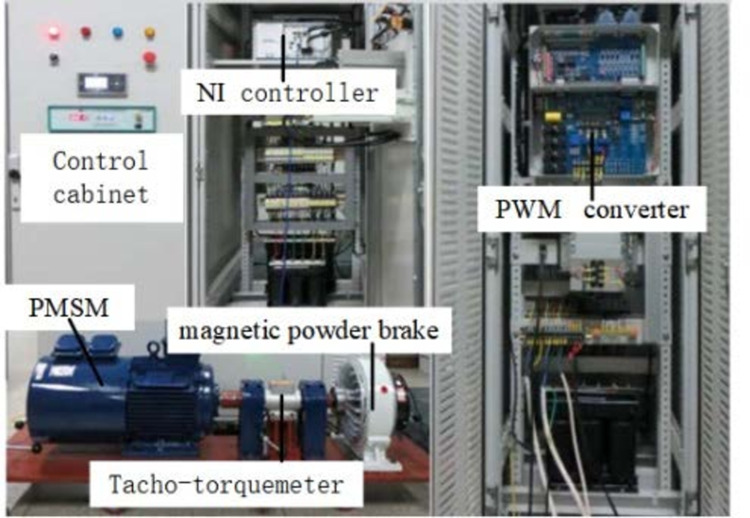
Experiment platform of PMSM control system.

In order to further verify the effectiveness of the method proposed in this paper, he experiment verification is carried out by using the comprehensive experimental platform of motor speed regulation and loading. The physical representation of the actual platform is shown in Fig 14.

The experimental platform primarily comprises the NI controller, inverter, speed and torque measuring instrument, PMSM, magnetic powder brake, monitoring PC, and essential power distribution lines. The proposed method designed in the Simulink environment is downloaded to the NI controller. Once the platform parameters are confirmed to be within normal range, the experiment on motor speed control is conducted. The real-time experimental curves, obtained through the monitoring PC, are analyzed to validate the feasibility and effectiveness of the proposed method as presented in this paper.

**Fig 15:**
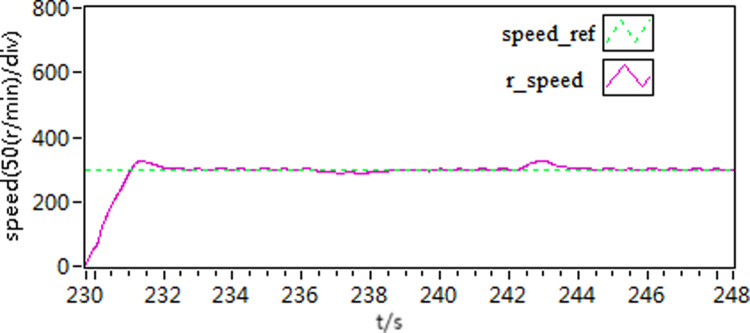
The speed curve of load variations by use of scheme 1.

**Fig 16:**
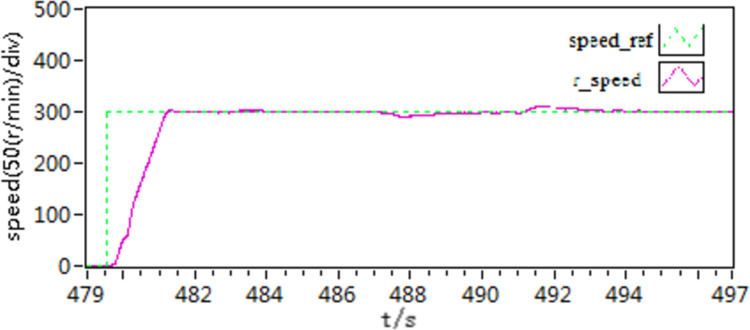
The speed curve of load variations by use of scheme 2.

**Fig 17:**
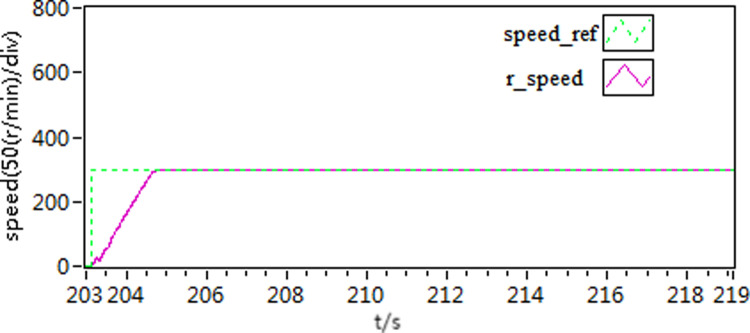
The speed curve of load variations by use of scheme 3.

**Fig 18:**
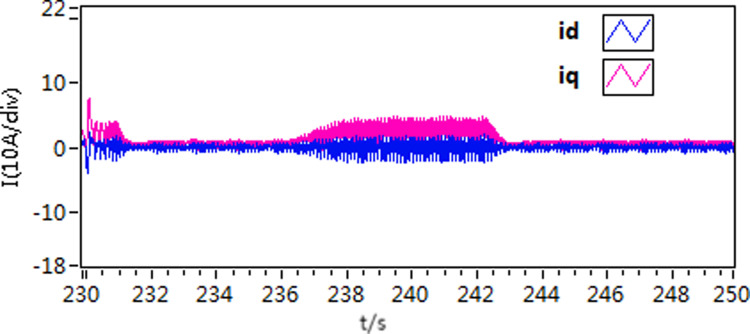
The d-q axis current by use of scheme 1.

**Fig 19:**
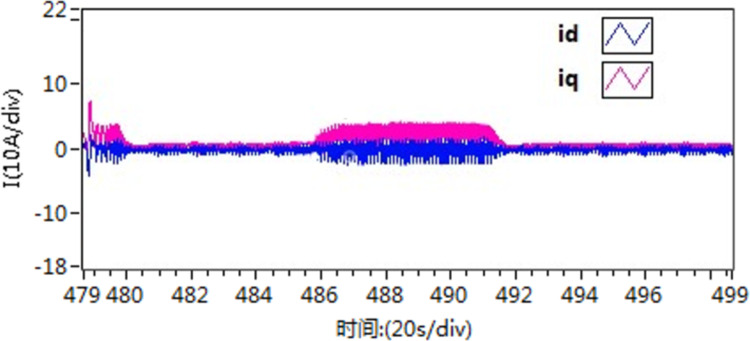
The d-q axis current by use of scheme 2.

**Fig 20:**
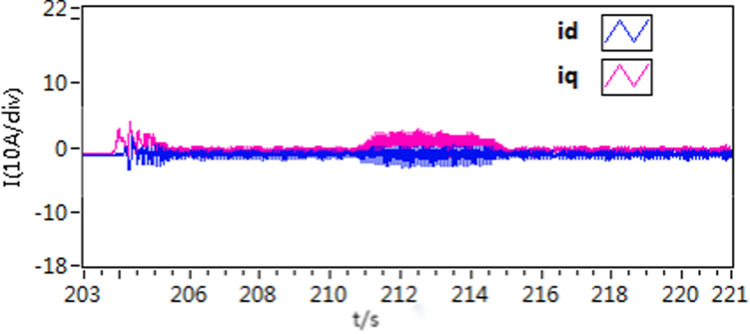
The d-q axis current by use of scheme 3.

In the first scenario, the motor speed is adjusted to 300 r/min. Once the motor achieves smooth operation, a torque load of 10 N**·**m is introduced. Subsequently, the load is subsequently removed after a certain period of running time. Figs 15–20 depict the motor speed curves and current curves for both control schemes. Upon comparing the motor speed curves between the three control schemes, it becomes evident that when the load undergoes changes, the speed fluctuation amplitude of scheme 1 is approximately 15%. Moreover, scheme 1 and scheme 2 exhibit a longer adjustment time in comparison to scheme 3.

Furthermore, upon comparing the present curves in response to variations in motor load, it becomes apparent that scheme 3 displays reduced current fluctuations and faster response throughout the loading and unloading procedures.

**Fig 21:**
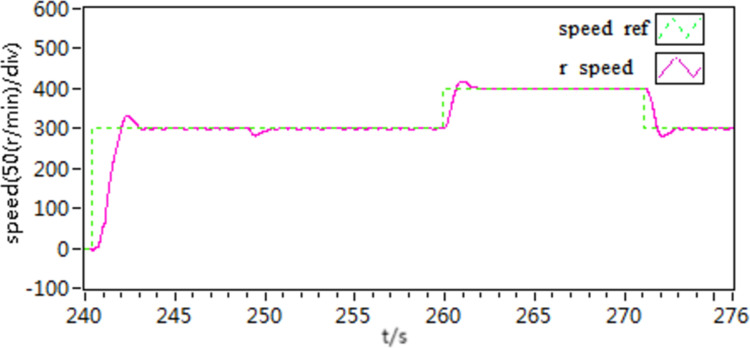
The speed curve of acceleration and deceleration experiment by use of scheme 1.

**Fig 22:**
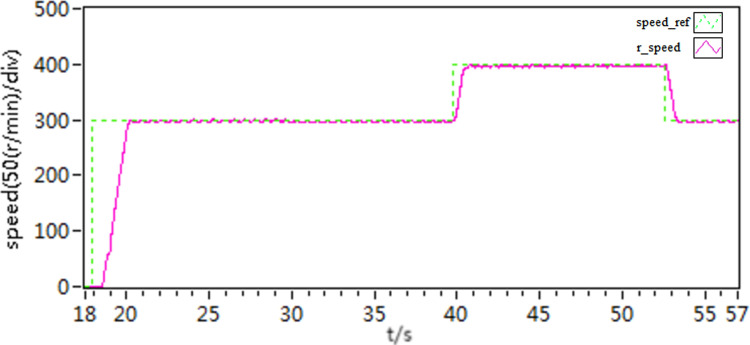
The speed curve of acceleration and deceleration experiment by use of scheme 2.

**Fig 23:**
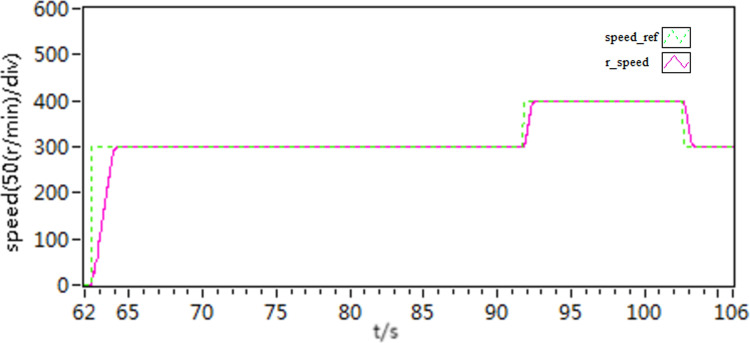
The speed curve of acceleration and deceleration experiment by use of scheme 3.

**Fig 24:**
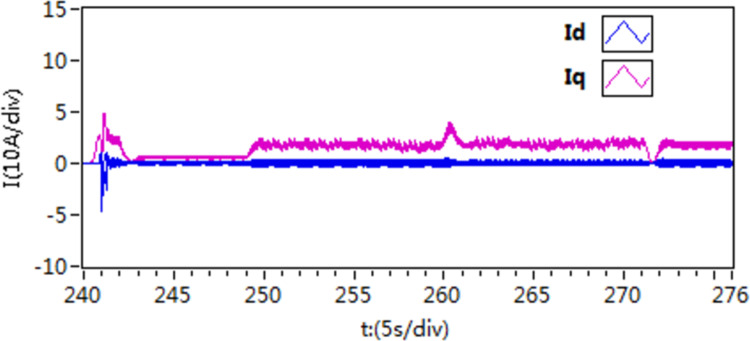
The d-q axis current by use of scheme 1.

**Fig 25:**
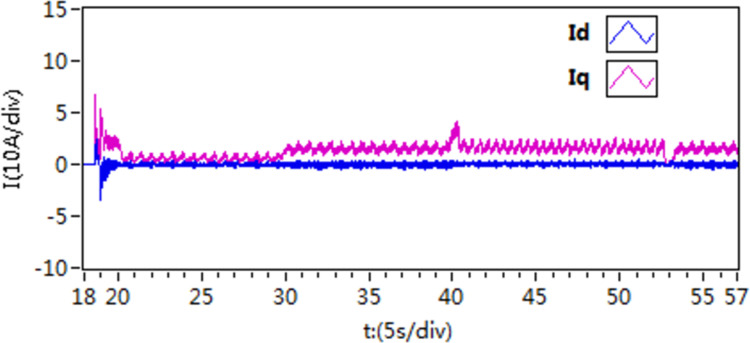
The d-q axis current by use of scheme 2.

**Fig 26:**
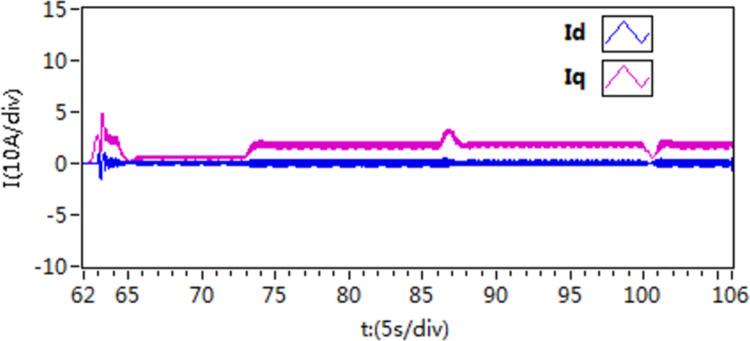
The d-q axis current by use of scheme 3.

In the second scenario, an experiment is conducted to observe the acceleration and deceleration of speed when the motor is loaded with torque. Initially, the motor is set to a speed of 300 r/min. Once the motor is started and running smoothly at this speed, then, the motor is loaded with a constant load torque of 10 N • m, it is then adjusted to 400 r/min. After a certain period, the speed is then readjusted back to 300 r/min. The recorded speed and current curves of this acceleration and deceleration experiment can be seen in Figs 21–26.

According to the Figs 21–26, it is concluded that scheme 3 is capable of achieving precise speed tracking without overshooting, irrespective of whether the motor is running at a higher speed or at a lower speed, and the current fluctuation of file scheme 3 is small, which makes it more resistant to interference.

**Fig 27:**
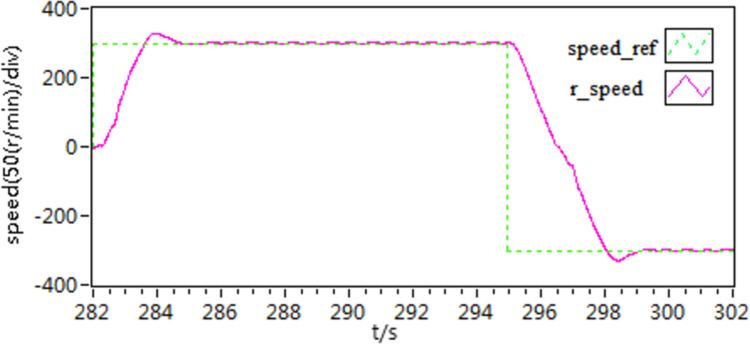
The speed curves of forward and reverse experiment by use of scheme 1.

**Fig 28:**
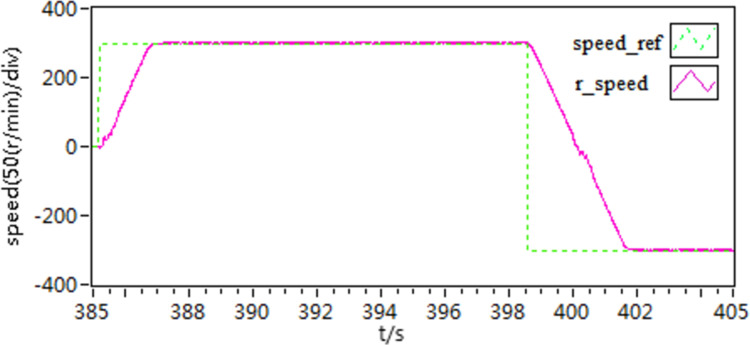
The speed curves of forward and reverse experiment by use of scheme 2.

**Fig 29:**
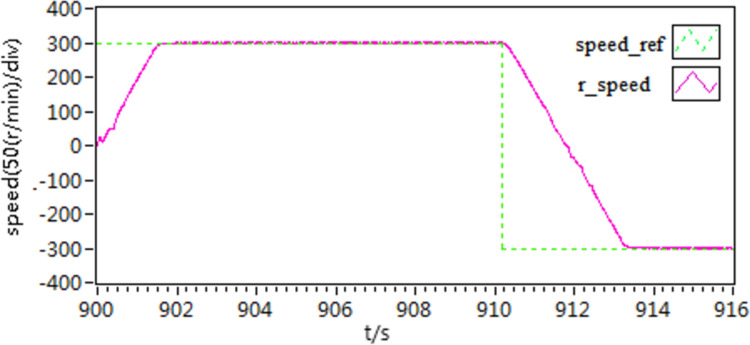
The speed curves of forward and reverse experiment by use of scheme 3.

**Fig 30:**
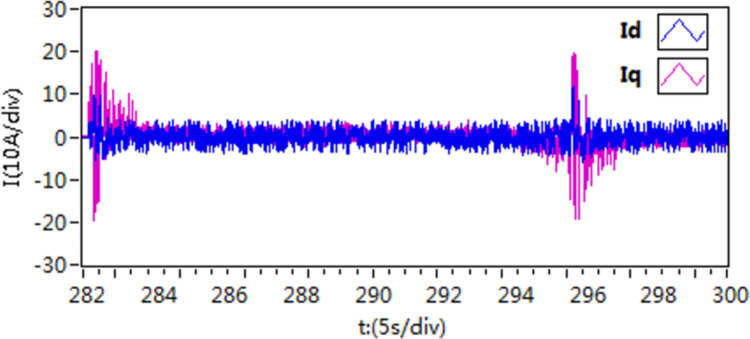
The d-q axis current curves of forward and reverse experiment by use of scheme 1.

**Fig 31:**
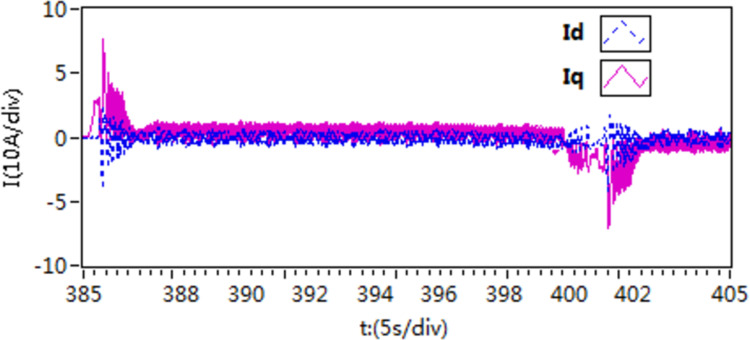
The d-q axis current curves of forward and reverse experiment by use of scheme 2.

**Fig 32:**
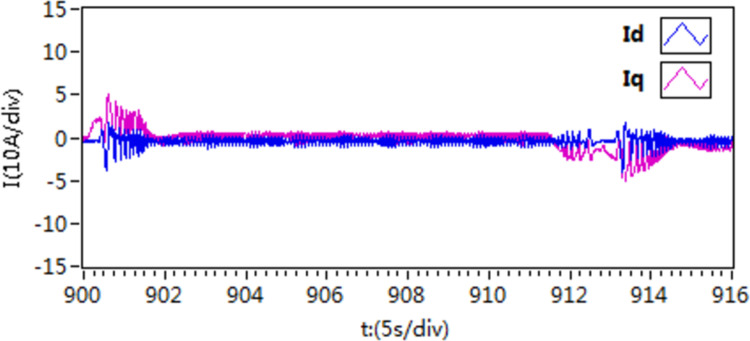
The d-q axis current curves of forward and reverse experiment by use of scheme 3.

In the third scenario, an experiment is conducted to test the motor’s ability to rotate in both forward and reverse directions. Initially, the motor speed is configured at 300 r/min, and then it is changed to -300 r/min. Figs 27–32 illustrate the results of this experiment. It is evident that when transitioning from forward to reverse rotation, scheme 1 shows a larger overshoot. In contrast, scheme 3 demonstrates the capability to rapidly adjust speed and smoothly transition, thereby demonstrating the robustness of the proposed control scheme.

## 5. Conclusion

This paper presents a novel methodology for the speed control system of PMSMs by integrating an improved GOA-based fuzzy PI speed controller with a MPCC in a cascaded structure. First, the inherent limitations of traditional GOA, such as susceptibility to local optima and premature convergence, are mitigated by incorporating mixed Cauchy variation and uniform distribution strategies. Subsequently, the enhanced GOA is applied to design a variable-domain fuzzy PI controller for the speed control loop, thereby enhancing the dynamic performance and robustness of the PMSM speed regulation system. Furthermore, to address the limitations of conventional PI control in the motor’s current control loop, a one-step-ahead MPCC is developed to minimize tracking errors and improve dynamic performance in the current loop. The proposed control strategy is rigorously validated through both simulations and experimental studies, demonstrating its effectiveness. Future research will focus on integrating disturbance estimation techniques into the proposed approach and addressing the computational burden associated with the control scheme to enhance its practical applicability.

## Supporting information

S1 Data Part of data. (ZIP)
